# Nootkatone, a Sesquiterpene Ketone From *Alpiniae oxyphyllae* Fructus, Ameliorates Metabolic-Associated Fatty Liver by Regulating AMPK and MAPK Signaling

**DOI:** 10.3389/fphar.2022.909280

**Published:** 2022-07-05

**Authors:** Zhang Yong, Huang Zibao, Zhou Zhi, Ma Ning, Wang Ruiqi, Chen Mimi, He Xiaowen, Dong Lin, Xia Zhixuan, Liu Qiang, Lu Weiying, Zhang Xiaopo

**Affiliations:** ^1^ Department of Pharmacology, Hainan Medical University, Haikou, China; ^2^ Key Laboratory of Tropical Translational Medicine of the Ministry of Education, Hainan Key Laboratory for Research and Development of Tropical Herbs, School of Pharmaceutical Science, Hainan Medical University, Haikou, China; ^3^ Reproductive Medical Center, Hainan Women and Children’s Medical Center, Haikou, China; ^4^ Public Research Laboratory, Hainan Medical University, Haikou, China

**Keywords:** MAFLD (metabolic-associated fatty liver disease), nootkatone, AMPK, MAPK, *Alpiniae oxyphyllae* Fructus

## Abstract

Metabolic-associated fatty liver disease (MAFLD) is becoming more common due to lifestyle changes. A long-term high-fat and high-glucose diet induces glycolipid metabolism disorders in the liver, which results in the development of MAFLD. To date, there is no specific clinically useful therapeutics for this disease. Natural products or synthetic compounds were screened and investigated to find effective agents for treating MAFLD. In this study, nootkatone (Nok), a natural sesquiterpene ketone isolated from *Alpiniae oxyphyllae* fructus, was explored for its potential to treat MAFLD, and underlying mechanisms were studied. Our results show that Nok dramatically ameliorated the disordered lipid and glucose metabolism in MAFLD mice, decreased fat accumulation in hepatic tissue, and improved liver injury. Inflammation, metabolic disorder, and oxidative stress were ameliorated in liver tissue based on RNA-seq transcriptome comparison between a Nok-treated group and an MAFLD model group. Furthermore, Nok significantly activated AMPK activity and inhibited MAPK activity*,* especially the p38 and JNK signaling pathways, *in vivo* based on western blot analysis. The pharmaceutical effects and potential signaling pathways impacted by Nok were also investigated in L02 cells. Nok significantly promoted the consumption of glucose and decreased the deposition of triglycerides *in vitro*. The p-AMPKα level was notably upregulated by Nok, indicating dramatic AMPK activation. In addition, Nok decreased the levels of p-ERK1/2, p-p38, and p-JNK. Nok also inhibited the activation of MAPK signaling and, thus, alleviated MAFLD development. Our results suggest that Nok may be useful in treating MAFLD. Nok may ameliorate MAFLD by regulating glycolipid metabolism disorders by activating AMPK and inhibiting MAPK activity. Collectively, this study suggests that Nok is an effective compound for the treatment of MAFLD.

## Introduction

With changing lifestyles, the incidence of nonalcoholic fatty liver disease (NAFLD) has increased. NAFLD has been newly termed metabolic-associated fatty liver disease (MAFLD) to exclude ethanol consumption and other liver disease factors in diagnosis (Eslam, Sanyal et al., 2020). MAFLD is the most common liver-related disease in the world and usually includes simple fatty liver, fatty liver hepatitis, liver fibrosis, alcoholic fatty liver, and even cirrhosis during development (Friedman, Neuschwander-Tetri et al., 2018; Eslam, Sanyal et al., 2020). MAFLD is becoming a great threat to human health, and the medical burden on society will increase with the increasing incidence (Younossi, Anstee et al., 2018). Multiple parallel hits produced in the liver from lipid accumulation, lipid toxicity from overaccumulation, oxidative stress and inflammation from lipid metabolic disorders, endoplasmic reticulum stress, mitochondrial damage, and insulin resistance are acknowledged as the main drivers of MAFLD pathogenesis (Younossi, Anstee et al., 2018; Eslam, Sanyal et al., 2020; Huang M, Kim H et al., 2020). Although there are many studies of MAFLD, the exact pathogenesis remains unclear, and there is no specific drug for treating MAFLD in the clinic (Kuchay, Choudhary et al., 2020). Finding effective and safe therapeutic agents from natural or synthetic resources for MAFLD is a common goal worldwide.

Glycolipid metabolism disorder in hepatocytes is considered a key development risk factor for MAFLD. Regulating hepatocyte glucose and lipid metabolism is beneficial to MAFLD and can inhibit the development of MAFLD. AMP-activated protein kinase (AMPK), the key enzyme regulating cellular glucose and lipid metabolism, is considered a cell energy sensor (Trefts and Shaw 2021). Targeting AMPK activation has been widely shown to correct glycolipid metabolism disorders and is useful for MAFLD treatment (Smith, Marcinko et al., 2016). The effects of AMPK activation in promoting glucose and lipid metabolic decomposition, inhibiting lipid synthesis and gluconeogenesis, and especially promoting a metabolic switch from fat synthesis to fat oxidation (Trefts and Shaw 2021) have been widely studied and reported. AMPK agonists are potential candidates for treating MAFLD.

Mitogen-activated protein kinase (MAPK) is also a very important kinase regulating a multitude of hepatic metabolic processes. There are three major MAPK subgroups in the mammalian liver, including extracellular signal-regulated kinases 1 and 2 (ERK1/2), c-Jun N-terminal kinases 1, 2, and 3 (JNK1/2/3), and p38α/β/δ/γ (p38) (Zeng, Tang et al., 2014; Lawan and Bennett 2017). MAPK is usually activated by disorders of glucose and lipid metabolism, oxidative stress, inflammation, and other factors associated with MAFLD (Lawan and Bennett 2017). Inhibiting the MAPK signaling pathway could improve MAFLD by regulating glucose and lipid metabolism and exhibiting anti-inflammatory, antioxidant, and other effects (Lawan, Zhang et al., 2015; Hwang, Wang et al., 2020). Many anti-MAFLD potential compounds, such as betaine (Ge, Yu et al., 2016), liraglutide (Zhang, Yang et al., 2013), and chlorogenic acid (Yan, Gao et al., 2018), inhibit MAPK activity. Suppressing MAPK signaling is a potentially effective therapeutic strategy for MAFLD treatment.

Nootkatone (Nok), 5,6-dimethyl-8-isopropyl-dicyclic-(4,4,0)-dec-1-ene-3-one, with a molecular formula of C_15_H_22_O, is a sesquiterpene ketone that naturally exists in *Alpiniae oxyphyllae* fructus, grapefruit, and citrus (Wang, Wang et al., 2018). *Alpiniae oxyphyllae* is a famous traditional Chinese medicine and Li Medicine, rich in Hainan Province of China. *Alpiniae oxyphyllae* has many effects in traditional Chinese medicine, including warming the spleen, stopping diarrhea, reducing salivation, warming the kidney, reducing urine, and solidifying essence (Li, Du et al., 2021). Previous studies have reported that Nok reduced weight gain (Murase, Misawa et al., 2010), increased the sensitivity of the non-small-cell cancer cell Line A549 to doxorubicin (Moon, Ryu et al., 2019), protected against chronic kidney injury (Li, Tan et al., 2016; Chen, Lin et al., 2021), and showed anti-anxiety and anti-depression effects (Yan, Li et al., 2021). To date, there have been no reports about its anti-MAFLD activity or potential mechanisms, which attracted our interest in this study.

## Materials and Methods

### Extraction and Isolation of Nok

Fructus (1.0 kg) from *Alpiniae oxyphyllae* was extracted using petroleum ether under reduced pressure (30 g) and further separated by a silica gel column eluted with a petroleum ether-ethyl acetate (1:0-0:1) gradient to afford six fractions (Fra. A-F). Fra. E was then isolated by Sephadex LH-20 to give Nok (20 mg). The purity of Nok was determined by HPLC. The methods were as follows. Column: Thermo HyPURITY C18, 4.6 × 150 mm, 3.0 μm; UV detector wavelength: 238 nm; mobile phase flow rate: 1.0 ml/min; sample: prepared in methanol; mobile phase: A, acetonitrile; B, 0.1% phosphoric acid in water; gradient elution conditions: A, 50%–60%, 15 min. To obtain a sufficient amount of the compound, 10 g of Nok was purchased from Chengdu Biopurify Phytochemicals Ltd., and its purity was also determined by HPLC.

### Animals and Treatments

A total of 32 male mice (age, 5 weeks; weight 19–21 g) were purchased from Gempharmatech Co., Ltd. (Jiang Su, China). Mice were fed in the animal research center of Hainan Medical University (HMU). Our study was approved by the ethics committee of HMU. The environmental surroundings were maintained at an indoor temperature of 25 ± 0.5°C with a 12-h light–dark cycle and free access to water and food.

After adaptive feeding for 3 days, the mice were stochastically divided, 8 mice each, into 4 experimental groups: normal, MAFLD model, 25 mg kg^−1^ Nok and 50 mg kg^−1^ Nok. The normal group was fed a basal control diet. The MAFLD model group was fed a 60% high-fat diet (60% fat, HFD). For the Nok test groups, mice were fed an HFD in addition to 25 mg kg^−1^·d^−1^ or 50 mg kg^−1^·d^−1^ Nok, i. g.; Nok was dissolved in vegetable oil. The same volume of vegetable oil was given to the normal group and MAFLD model group mice. Fasting blood glucose levels and body weights were tested weekly during the experimental period. After 12 weeks of drug intervention, blood samples were taken after 8 h of fasting, and serum samples were obtained by centrifugation for further testing. Then, the mice were killed by CO_2_, and the liver tissues were used for other experiments.

### Oral Glucose Tolerance Test

During the 11–12 weeks of administration, the OGTT was performed as follows. The blood glucose level of each mouse was tested after 8 h of fasting overnight, and the data were defined as an initial blood glucose value of 0 min. Each mouse was orally gavaged with 50% glucose solution, and the volume was calculated according to 0.02 g/10 g. We then detected blood glucose levels using a glucose meter (Roche, ACCU-CHEK) 30, 60, 120, and 180 min after oral glucose administration.

### Serum Biochemical Analysis

After 12 weeks of drug administration, blood was taken, and the serum was isolated by centrifugation at 3,000 rpm at 15°C for 15 min. Then, serum lipids, such as triglyceride (TG), low-density lipoprotein cholesterol (LDL-c), high-density lipoprotein cholesterol (HDL-c), and total cholesterol (TC), were tested by appropriate kits (Nanjing Jiancheng Bioengineering Institute). The hepatic function indices in serum, such as alanine aminotransferase (ALT) and aspartate aminotransferase (AST), were also measured using kits (Nanjing Jiancheng Bioengineering Institute). The serum IL-1β, TNFα, IL-6, and IL-18 levels were assayed by corresponding ELISA kits from Nanjing Jiancheng Bioengineering Institute.

### Liver Histopathological Analysis

After 12 weeks of drug administration, liver tissue was isolated from the same part of the mouse liver and drenched in a 10% formalin tissue fixator for 48 h. After sufficient tissue fixation, liver samples were embedded in paraffin, sectioned (4 μm), stained with hematoxylin and eosin (H&E), and imaged. Oil Red O staining was also performed on liver tissues to detect lipid deposition. Scores of hepatic tissue steatosis based on H&E staining were used to evaluate the pathological changes, and 5 grades were defined as follows: 0%–5% of parenchyma-involved steatosis was scored as zero, 5%–25% was scored as one, 25%–50% was scored as two, 50%–75% was scored as three, and over 75% was scored as four. Oil Red O staining was quantified by the red area coefficient.

### RNA-Seq Assay

Three mouse hepatic tissues were isolated from the normal, MAFLD model, and Nok (50 mg kg^−1^) treatment groups. Total RNA was extracted, and quality was assessed by integrity. Relevant sample processing, sequencing, and data analysis methods were described in our previous research (Yong, Ruiqi et al., 2021).

### Cell Culture and Treatment

L02 hepatocytes were used in this study, and the culture medium was DMEM supplemented with 10% fetal bovine serum (FBS) and 1% penicillin–streptomycin solution. After two generations of stable passage of L02 cells, the cell suspension was prepared and seeded into the plates and cultured for approximately 24 h. Nok and metformin (Met) dissolved in dimethylsulfoxide (DMSO) were added and treated with or without PO (200 μM PA-BSA and 400 μM OA-BSA) coincubation for another 24 h. For blocking experiments, L02 cells were cultured and seeded as above, and before drug treatment, the cells were pretreated with a AMPK inhibitor Compound C (CC, dissolved in DMSO) at 10 μM or a JNK agonist anisomycin (AN, dissolved in DMSO) at 1 μM for 1 h. Following pretreatment, PO and drugs were added to the corresponding wells and cocultured with the cells for 24 h. Then, total cellular protein was extracted after treatment and detected by western blotting.

### Cytotoxicity Assay

The cytotoxicity of Nok to L02 cells was evaluated by the MTT method. Cell culture and treatment were performed as above, and 5 replicates were performed for each drug treatment. The highest treatment concentration of Nok was 400 μM. A similar volume of DMSO (volume ratio as 0.1%) was added to the control group. After 24 h of drug treatment, the culture medium of each well was removed, and 50 μl MTT solution was added to the cells. Then, the plates were incubated at 37°C for 4 h. The level of formazan was detected at OD 490 nm with a microplate reader to assess cell viability. The DMSO treatment group was normalized to 100% cell viability, and the others are shown as percentages of the DMSO treatment group.

### Glucose Consumption Assay

Cell culture and treatment were performed as above, and 5 replicates were performed for each drug treatment. The glucose consumption was calculated by subtracting the glucose level present in media from cells cultured for 24 h with drug or equal volume DMSO (volume ratio as 0.1%) treatment from the glucose level in plates containing only culture medium. After drug treatment, the glucose concentration in the supernatant of each cell culture well was measured by a glucose detection kit (Beijing Strong Biotechnologies, Inc.), and the glucose consumption level of each treatment group was calculated as above.

### Intracellular TG Assay

Cell culture was performed as described above. L02 cells were inoculated in a cell culture plate at a density of 1.0*10^5^ cells/ml, and four replicates were performed for each drug treatment. Then, 600 μM PO (200 μM PA-BSA and 400 μM OA-BSA) was added to induce a cell model of intracellular TG accumulation. The normal control group was supplemented with 10% BSA solution. After incubation with PO or BSA for 24 h, equal volumes of DMSO (volume ratio of 0.1%) to 40 μM Nok, or Nok or Met at the indicated concentrations were added to the cell culture wells for another 24 h of incubation. The cells were collected, the intracellular TG content was determined by a cellular TG assay kit (Applygen Technologies Inc.), and the intracellular TG level was normalized to the protein concentration. With the same treatment, the oil red O staining was done to evaluate the intracellular lipid accumulation induced by PO.

### Intracellular ATP Assay

With the same treatment conditions as intracellular TG assay, the intracellular ATP in L02 cells was detected by ATP assay kits.

### Western Blots

The L02 cells and liver tissue samples were prepared as described above, and total protein was extracted. Western blotting was used to detect the target proteins of each sample, such as phospho-ERK1/2 (p-ERK1/2) (AM071, Beyotime), ERK1/2 (AF1051, Beyotime), phospho-p38 MAPK (Thr180) (p-p38) (AF5884, Beyotime), p38 MAPK (p38) (AF7668, Beyotime), phospho-JNK1/2 (Thr183/Tyr185) (p-JNK) (AF5860, Beyotime), JNK (AJ518, Beyotime), GAPDH (AF1186, Beyotime), phospho-AMPKα (Thr172) (p-AMPKα) (2535T, CST), AMPKα (t-AMPK) (2532S, CST), phospho-ACC (Ser79) (p-ACC) (3661S, CST), and ACC (t-ACC) (3676T, CST). The target protein bands of western blots were analyzed and quantified. The levels of p-AMPKα, p-ACC p-ERK1/2, p-p38 and p-JNK were normalized to those of AMPKα, ACC, ERK1/2, p38 and JNK, respectively, and are presented as the fold change compared to the control treatment.

### Quantitative PCR Analysis

Total RNA was extracted from liver tissues, and quantitative PCR (qPCR) assays were performed with SYBR Green PCR master mix (rr42lr, Takara, Beijing, China) in an ABI Prism 7,900 high-throughput real-time PCR system. The forward (F) and reverse (R) primer sequences were as follows: Il-1β (F, 5′cga​caa​aat​acc​tgt​ggc​ct3’; R, 5′ttc​ttt​ggg​tat​tgc​ttg​gg3′), IL-6 (R, 5′tagtcc ttc​cta​ccc​caa​ttt​cc3’; R, 5′ttg​gtc​ctt​agc​cac​tcc​ttc3′), TNFα (F, 5′ccc​tca​cac​tca​gat​cat​ctt​ct3’; R, 5′gct​acg​acg​tgg​gct​aca​g3′), IL-18 (F, 5′cat​gcc​atg​gct​gct​gaa​cca​gta​gaa​ga3’; R, 5′cgggatcc aat​agc​tag​tct​tcg​ttt​tg3′) and β-actin (F, 5′gga​tgc​aga​agg​aga​tta​ctg​c3’; R, 5′ccaccg atccacagagta3′).

### Statistical Analysis

For *in vitro* experiments, our values are displayed as the mean ± SD with three independent repetitions. For *the in vivo* experiments, our results are also presented as the mean ± SD. There were 8 mice in each group. GraphPad Prism 5.0 software was used to process and analyze data. The differences among the studied groups were assessed by *t* test or one-way ANOVA, and *p* < 0.05 was considered statistically significant.

## Results

### Extraction and Identification of Nok

**FIGURE 1:**
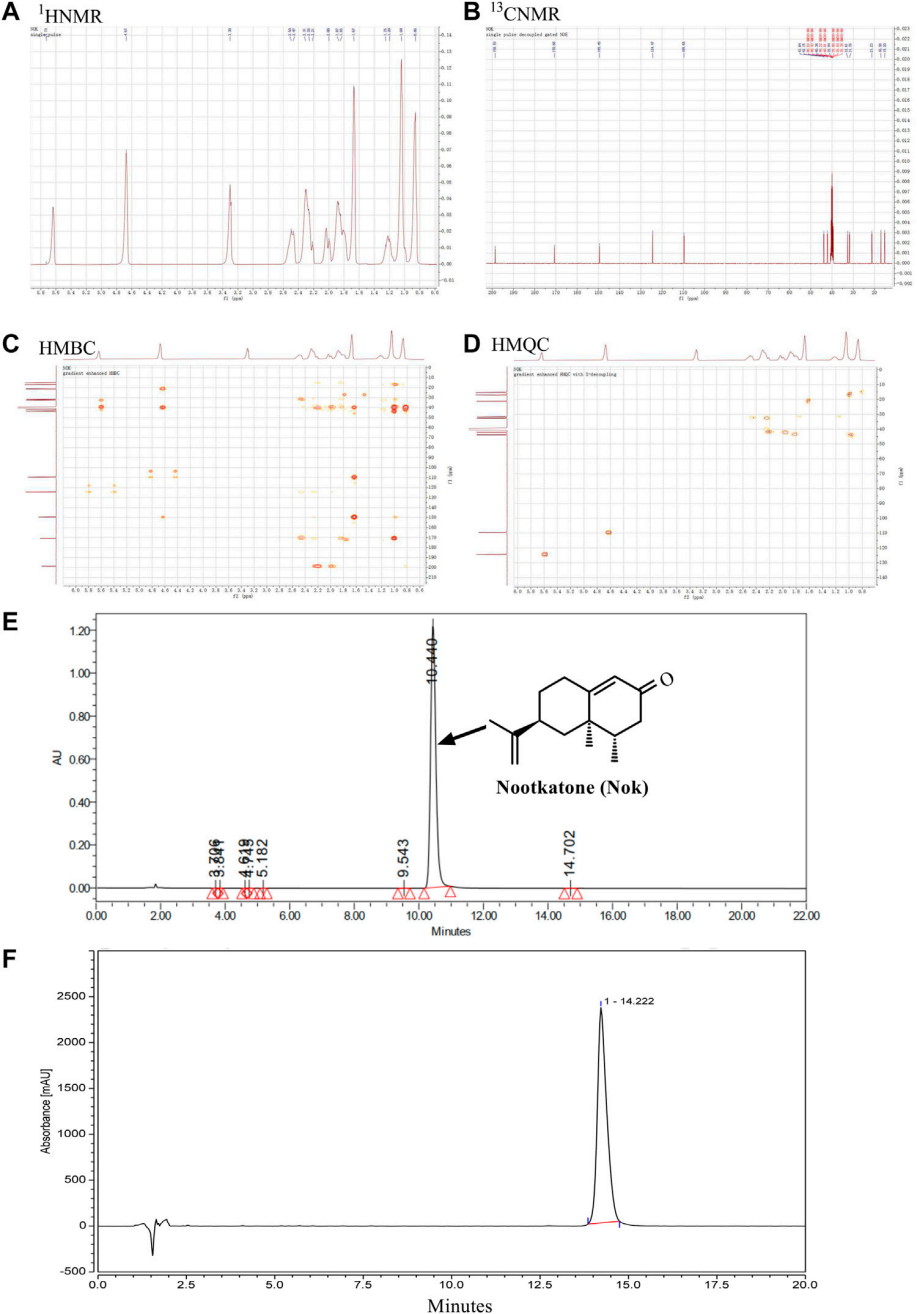
Isolation and structural characterization of nootkatone. **(A)** The structure and ^1^H-NMR spectrum of Nok **(B)** ^13^C-NMR spectrum of Nok; **(C)** HMQC spectrum of Nok **(D)** HMBC spectrum of Nok. **(E)** HPLC of Nok from *Alpiniae oxyphyllae* Fructus. **(F)** HPLC of Nok from Chengdu Biopurify Phytochemicals Ltd.

**FIGURE 2:**
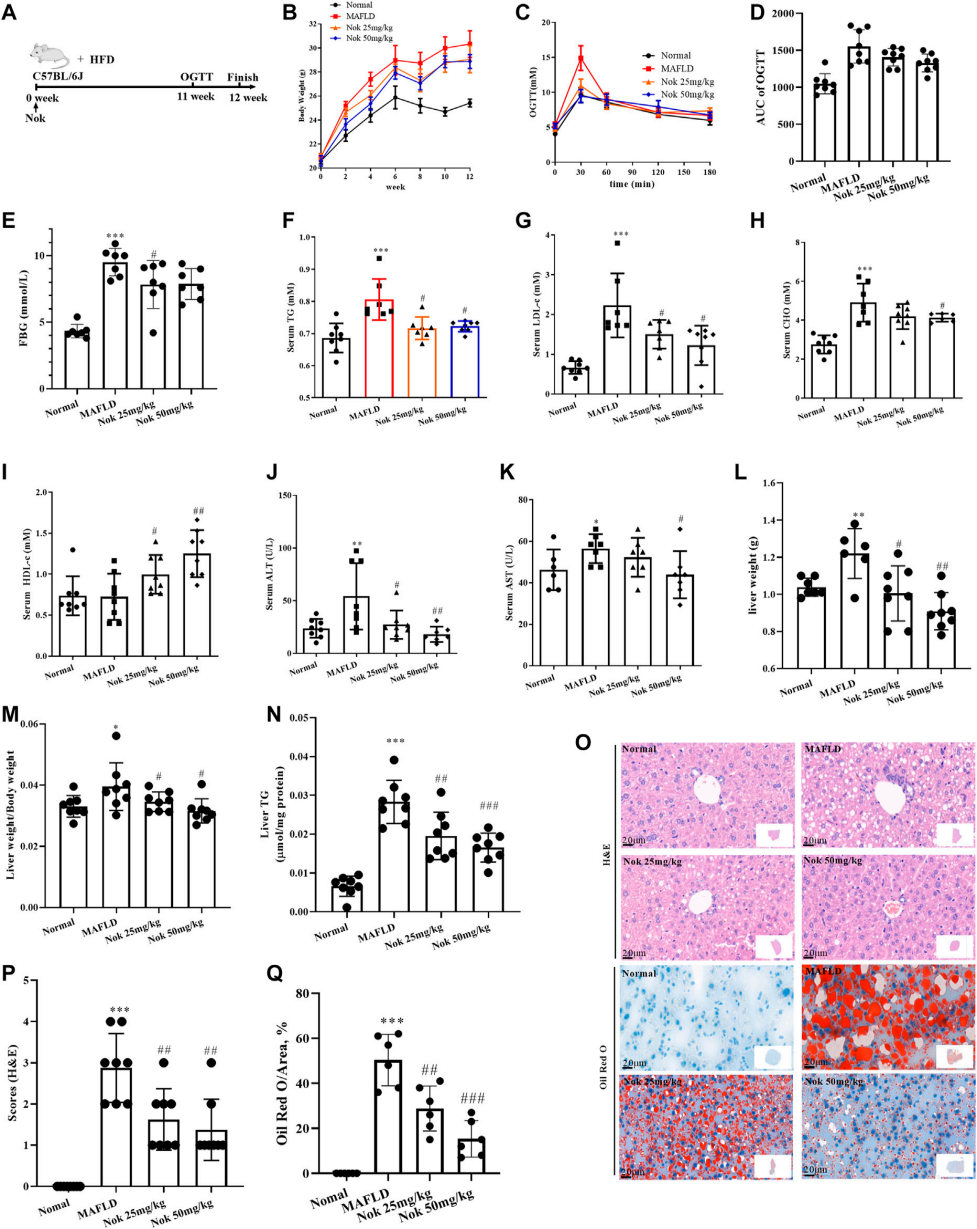
Nootkatone ameliorated metabolic-associated fatty liver. Mice fed a high-fat diet were administered Nok at doses of 25 mg kg^−1^ and 50 mg kg^−1^ daily for 12 weeks **(A)**. Changes in body weight **(B)**, OGTT test results at 11–12 weeks **(C)** and OGTT AUC **(D)**, FBG **(E)**, serum lipid **(F–I)**, liver function **(K–L)**, liver index (liver weight/body weight, **(M)**, liver TG **(N)**, and liver histopathological examination including H&E and Oil Red staining **(O)**, grading **(P)** and quantity by red area coefficient **(Q)** were measured. All data points represent the means ± SD (n = 8 per group); *p < 0.05, **p < 0.01, ***p < 0.001 vs. normal group mice; #*p* < 0.05, ##*p* < 0.01, ###*p* < 0.001 vs. MAFLD group mice.

Nok was identified by analyzing NMR data. The ^1^H-NMR (400 MHz, DMSO-*d*_*6*_) spectrum displayed signals of *δ*_H_: 5.64 (1H, brs), 4.67 (2H, brs), 1.66 (3H, brs), 1.04 (3H, s), 0.87 (3H, d, *J* = 7.2 Hz) (Figure 1A), which were attributed to two double bonds and three methyl groups. In the ^13^C-NMR spectrum (100 MHz, DMSO-*d*_*6*_), fifteen carbon signals were observed (Figure 2B). These carbon signals were *δ*_C_: 198.6, 170.1, 149.5, 124.5, 109.7, 43.8, 42.1, 40.5, 40.4, 39.9, 32.6, 31.7, 21.2, 17.0, and 15.2 (Figure 2B). These NMR data were identical to Nok and were further confirmed by analyzing 2D HMQC and HMBC NMR spectra (Figures 1C,D). In the HMQC spectrum, correlations between hydrogen and carbons were observed to assign the CH, CH_2_, and CH_3_ in Nok. In the HMBC spectrum, long-range correlations between hydrogen and carbons were carefully analyzed for connecting different structural units together to confirm the structure of Nok. The purity of Nok from our extraction and a commercial source were greater than 99% based on HPLC (Figures 1E,F).

### Nok Ameliorates MAFLD Effects

The timeline and design of the animal experiments are shown in Figure 2A. The body weight (Figure 2B), OGTT and AUC (Figures 2C,D), levels of fasting blood glucose (Figure 2E), serum TG (Figure 2F), LDL-c (Figure 2G) and CHO (Figure 2H) of the MAFLD model group mice were prominently higher than those of the normal control group, and HDL-c was decreased (Figure 2I), indicating significant glucose and lipid metabolism disorders. At the same time, the liver weight (Figure 2L), liver index (Figure 2M) and liver TG (Figure 2N) of mice in the MAFLD group were also significantly increased, and liver function (Figures 2G–K) and liver glucose tolerance (Figures 2C,D) were impaired. The H&E and oil red staining (Figure 2O) results showed that the model group displayed significant fatty liver characteristics, including bullae steatosis, inflammation and lipid accumulation. Meanwhile, the steatosis score (Figure 2P) and oil red O-positive area (Figure 2Q) were significantly higher than those of the normal group, suggesting that the model mice had significant metabolism-associated fatty liver disease. In the Nok-treated group, body weight (Figure 2B), fasting glucose (Figure 2E), and blood lipid levels (Figures 2F–H) were significantly reduced, serum HDL-c levels (Figure 2I) and glucose tolerance conditions (Figures 2C,D) were significantly improved, and liver function was recovered (Figures 2G–K). The liver weight (Figure 2L), liver index (Figure 2M) and liver TG (Figure 2N) were significantly decreased. Histopathological changes were significantly ameliorated in the Nok treatment group mice compared with the MAFLD model group (Figure 2O–Q). This abnormality in the MAFLD model was dose-dependently improved by Nok treatment (Figure 2B-Q). Collectively, these results suggested that Nok had the capability to improve MAFLD.

### RNA-Seq Assay of the Effects of Nok on MAFLD Treatment

**FIGURE 3:**
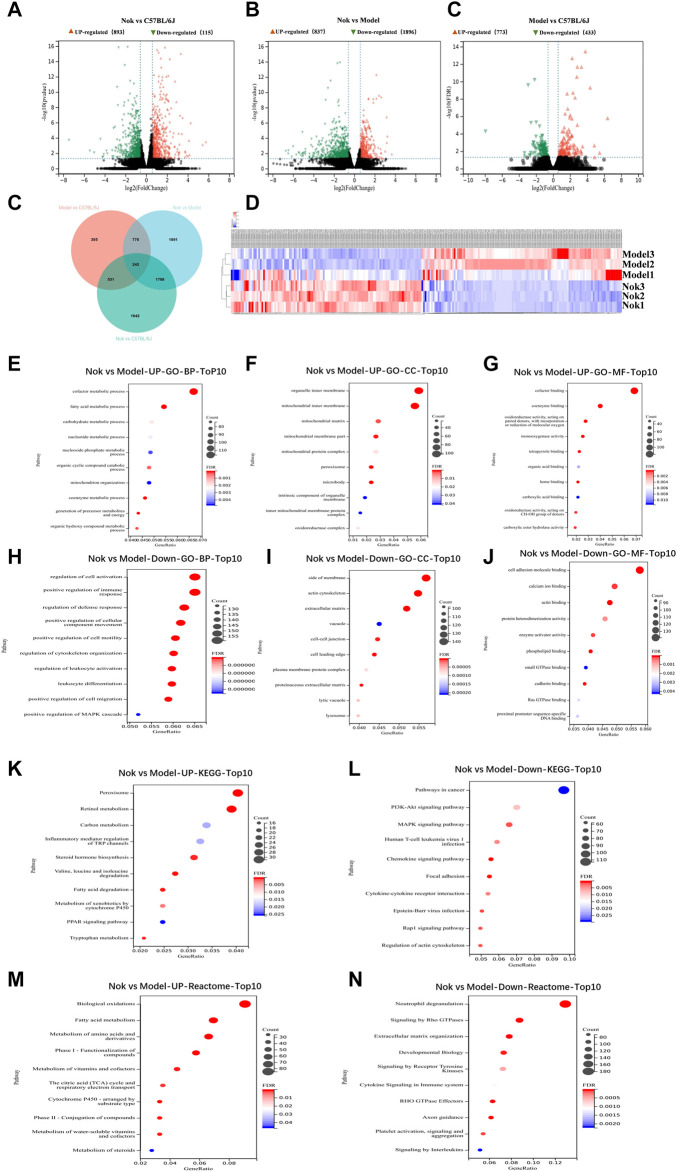
Analysis of hepatic transcriptome**.** RNA-seq assay of mouse hepatocytes between the Nok treatment group (n = 3) and MAFLD model group (n = 3). Volcano plots show the differentially expressed genes in **(A)** Nok-treated vs. normal mice, **(B)** Nok-treated vs. MAFLD mice, and **(C)** MAFLD vs. normal mice show fold changes (|log_2_ (fold change)|>0.58 and adjusted *p* value padj≤0.05). **(D)** Venn diagram showing the intersections among differentially expressed genes in different group-to-group comparisons. **(E)** Heatmap showing the differentially expressed genes between Nok-treated and MAFLD model mice. **(F–K)** Gene Ontology (GO) analysis showing the main changes in gene functions between Nok-treated and MAFLD model mice. **(L–M)** Kyoto Encyclopedia of Genes and Genomes (KEGG) showing enriched differentially expressed genes belonging to different pathways between Nok-treated and MAFLD model mice. **(N–O)** The Reactcomes of the main changes of gene functions between Nok treated and MAFLD model mice.

RNA-seq of liver tissue was performed to investigate the mechanisms underlying Nok’s effects on MAFLD. In the normal group, the MAFLD model group, and the Nok (50 mg kg^−1^·d^−1^) treatment group, 3 mouse liver tissues from each group were isolated, and total RNA was extracted. In the MAFLD group, compared with the normal group, there were 773 upregulated genes and 433 downregulated genes (Figure 3C). In the Nok (50 mg kg^−1^)-treated group, compared with the normal group mice, there were 893 upregulated genes and 1,153 downregulated genes (Figure 3A), and compared to the MAFLD model group mice, there were 837 upregulated genes and 1,896 downregulated genes (Figure 3B). All changes were based on an FDR<0.05 and fold change>1.5. These three groups of differentially expressed genes are depicted in the Venn diagram in Figure 3D; 242 genes are common to all three comparisons, including 119 upregulated genes and 123 downregulated genes in the Nok group (Figures 3D,E). These genes were visualized by a heatmap (Figure 3E). Gene ontology (GO) enrichment was analyzed by the clusterProfiler R package and sangerbox, and GO terms with padj less than 0.05 were considered significantly differentially enriched. The biological processes (BP), cellular components (CC), and molecular functions (MF) of significantly upregulated genes in the Nok treatment group compared to the MAFLD model group mice are shown in Figures 3F–H. These were mainly related to influencing metabolic processes, fatty acid metabolic processes and carbohydrate metabolic processes in BP and were mainly associated with the organelle inner membrane, mitochondrial matrix, mitochondrial membrane, and mitochondrial protein complex in CC and cofactor binding and coenzyme binding in MF (Figures 3F–H). The biological processes (BP), cellular components (CC), and molecular functions (MF) of significantly downregulated genes in the Nok treatment group compared to the MAFLD model group mice are shown in Figures 3I–K. These genes were mainly associated with positive regulation of the immune response, defense response, cell motility, leukocyte activation, and MAPK cascade in BP and were also correlated with side of membrane, actin cytoskeleton, extracellular matrix, vacuole, cell–cell junction, cell leading edge, plasma membrane protein components in CC and cell adhesion molecule binding, actin binding, protein heterodimerization activity, enzyme activator activity, phospholipid binding, calcium ion binding, and small GTPase binding in MF (Figures 3I–K). These results suggested that Nok might act on the immune system and that its pharmacological effects might be related to inflammation. The clusterProfiler R package was applied to assay the differential expression gene enrichments in KEGG pathways for the Nok treatment group compared to the MAFLD model group mice. The upregulated gene enrichment results showed that Nok could significantly promote peroxisome, retinol metabolism, carbon metabolism, steroid hormone biosynthesis, and fatty acid degradation signaling pathways (Figure 3L). The downregulated gene enrichment pathways suggested that Nok could significantly inhibit the PI3K-AKT, MAPK, chemokine, and Rap1 signaling pathways (Figure 3M). The reactome assay also showed that Nok may regulate biological oxidation, fatty acid metabolism and inflammation in the liver (Figure 3N–O).

### Nok Cytotoxicity in L02 Cells

**FIGURE 4:**
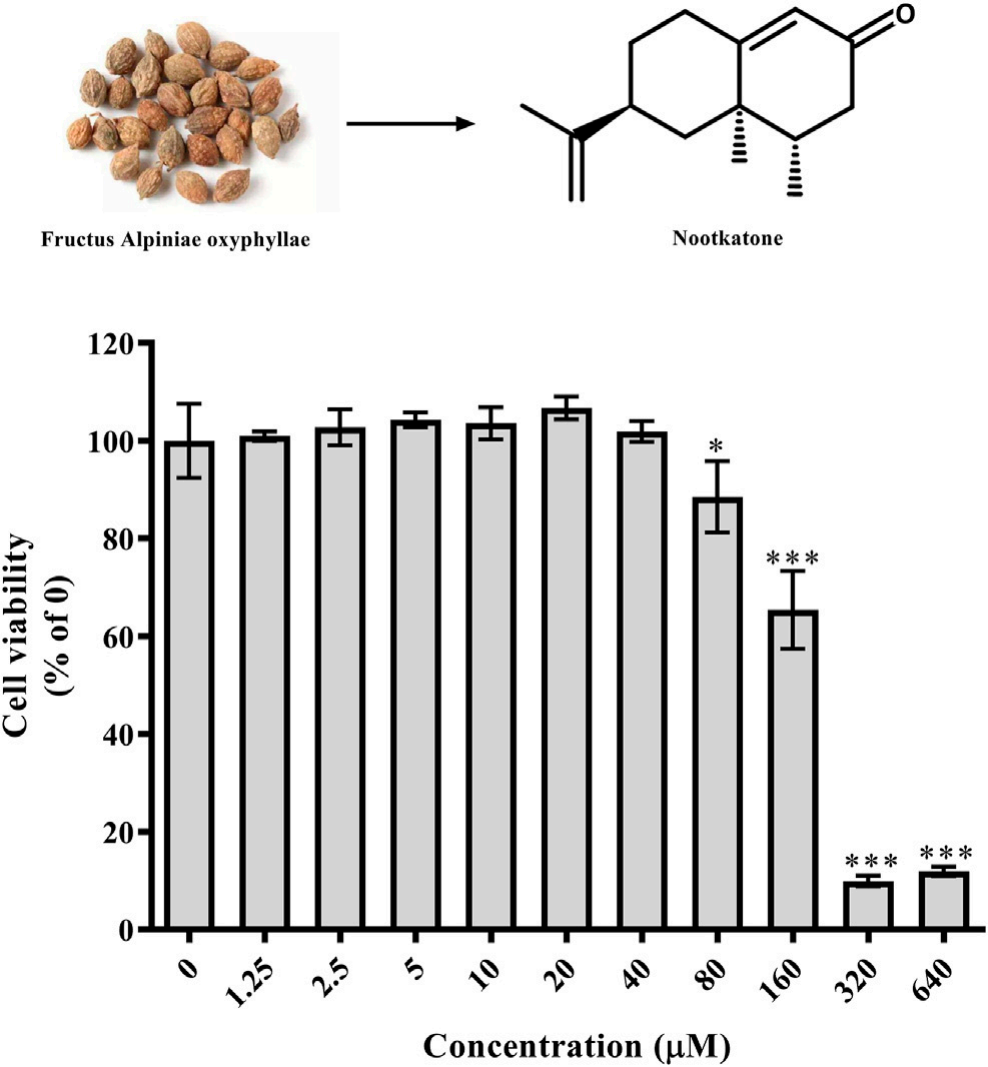
Effects of Nok on cell viability *in vitro*. L02 cells were incubated with DMSO or Nok at the corresponding concentration for 24 h, and viability was tested by MTT staining. All values are presented as the means ± SD, *p < 0.05, **p < 0.01, ***p < 0.001 vs. DMSO (0).

As shown in Figure 4, Nok showed no cytotoxicity at a concentration of 40 μM in L02 cells. However, the viability of L02 cells was significantly reduced at 80 μM (Figure 4, *p* < 0.05 vs. DMSO).

### Nok Regulates Hepatocyte Glucose and Lipid Metabolism by AMPK Activation *In Vitro* and *In Vivo*

**FIGURE 5:**
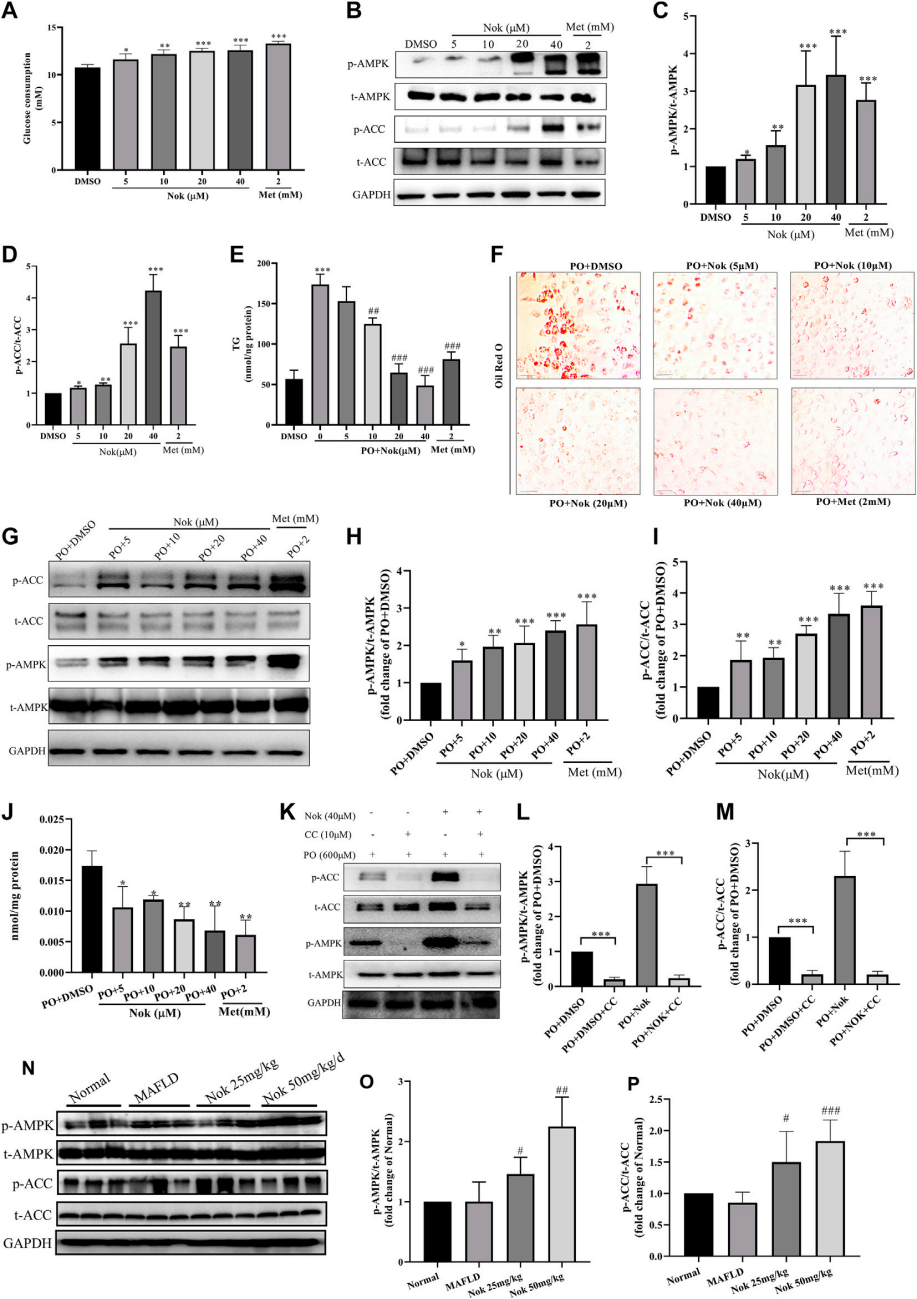
The effects of Nok on glucose and lipid metabolism and AMPK activation *in vitro* and *in vivo*. **(A)** Glucose consumption; values are the means ± SD, n = 5, *p < 0.05, **p < 0.01 or ***p < 0.001 vs. DMSO. **(B)** Western blots were performed to analyze the levels of p-AMPK, AMPK, p-ACC and ACC in L02 cells. Examples of representative blots as above and fold changes of p-AMPK/T-AMPK **(C)** and p-ACC/T-ACC **(D)** are shown by semiquantitative analyses. Values are the means ± SD from three separate experiments, *p < 0.05, **p < 0.01, ***p < 0.001 vs. DMSO. **(E)** Intracellular TG detection in L02 cells treated with Nok and undergoing PO induction. Values are the means ± SD, n = 4, ***p < 0.001 vs. PO + DMSO, ##*p* < 0.01 or ###*p* < 0.001 vs. PO + DMSO. (O) Oil Red O staining detection in L02 cells treated with Nok and undergoing PO induction, and the representative image as above. **(G)** Western blots were performed to analyze the phosphorylated and total protein levels of AMPK and ACC in L02 cells with Nok treatment and PO induction, and representative western blot images are shown above. The fold changes in p-AMPK/T-AMPK **(H)** and p-ACC/T-ACC **(I)** are shown by semiquantitative analyses. The values are the means ± SD from three separate experiments, *p < 0.05, **p < 0.01, ***p < 0.001 vs. PO + DMSO. **(J)** Intracellular ATP detection in L02 cells treated with Nok and undergoing PO induction. Values are the means ± SD, n = 3, *p < 0.05, **p < 0.01 vs. PO + DMSO. **(K)** Inhibitory effect of Compound C (CC) on Nok activation of AMPK. Representative western blot images are shown, and fold changes in p-AMPK/T-AMPK **(L)** and p-ACC/T-ACC **(M)** are shown by semiquantitative analyses. Values are the means ± SD from three separate experiments, ***p < 0.001. After 12 weeks of Nok treatment of C57BL/6J mice with a HFD, total protein was extracted from the livers. The p-AMPK, t-AMPK, p-ACC, t-ACC and GAPDH levels in liver tissues were determined by western blotting. Representative blots for each group are presented in **(N)**, and fold changes in p-AMPK/t-AMPK **(O)** and p-ACC/t-ACC **(P)** are depicted above. Values are the means ± SD (n = 6 per group); #*p* < 0.05, ##*p* < 0.01, ###*p* < 0.001 vs. MAFLD group mice.

As shown in Figures 5A, 5 μM Nok significantly increased glucose consumption (Figure 5A, p ˂ 0.05 vs. DMSO), and the glucose consumption-promoting activity of 40 μM Nok was similar to that of metformin at 2 mM (Figure 5A). Moreover, Nok exhibited a remarkable dose-dependent increase in glucose consumption in L02 cells (Figure 5A, p ˂ 0.05, p ˂ 0.01 or p ˂ 0.001 vs. DMSO). Western blot analysis showed that Nok activated AMPK (Figure 5B) and dose-dependently increased the levels of p-AMPKα (Thr172) and p-ACC (Ser79) in L02 cells (Figures 5B–D, p ˂ 0.05 or p ˂ 0.01 or p ˂ 0.001 vs. DMSO). We further investigated the effects of Nok on lipid metabolism through a PO-induced hepatocyte lipid accumulation model *in vitro*. As shown in Figure 5E, the levels of intracellular TG in L02 cells were dramatically increased by incubation with 0.6 mM PO (0.2 mM PA-BSA and 0.4 mM OA-BSA) for 24 h (Figure 5E, p ˂ 0.001 vs. DMSO+10% BSA), but they were dose-dependently reduced by the addition of Nok (Figure 5E, p ˂ 0.01 or p ˂ 0.001 vs. PO + DMSO). The oil red O staining results showed that more lipid accumulation was observed in L02 cells induced by PO (Figure 5F), but Nok treatment limited this accumulation (Figure 5F). These phenomena showed that Nok could regulate glycolipid metabolism in L02 cells (Figures 5E,F). Because AMPK plays a key role in the regulation of intracellular energy metabolism and glycolipid metabolism, we tested the effects of Nok on AMPK signal pathway activation in L02 cells undergoing PO treatment. Nok could also activate the AMPK signaling pathway (Figure 5G), and dose-dependently increased the levels of p-AMPKα (Thr172) and p-ACC (Ser79) in L02 cells treated with PO (Figures 5H,I). Nok also decreased the production of ATP in L02 cells with PO incubation (Figure 5J). Moreover, AMPK activation was abolished by pretreatment with Compound C (CC), an AMPK inhibitor (Figure 5K-M). The AMPK activation of Nok was also observed by western blots of liver tissues (Figure 5N–P). These results suggest that the effects of Nok on hepatocyte glucose and lipid metabolism regulation may be dependent on AMPK activation (Figure 5).

### Nok Inhibits MAPK Signaling in Hepatocytes

**FIGURE 6:**
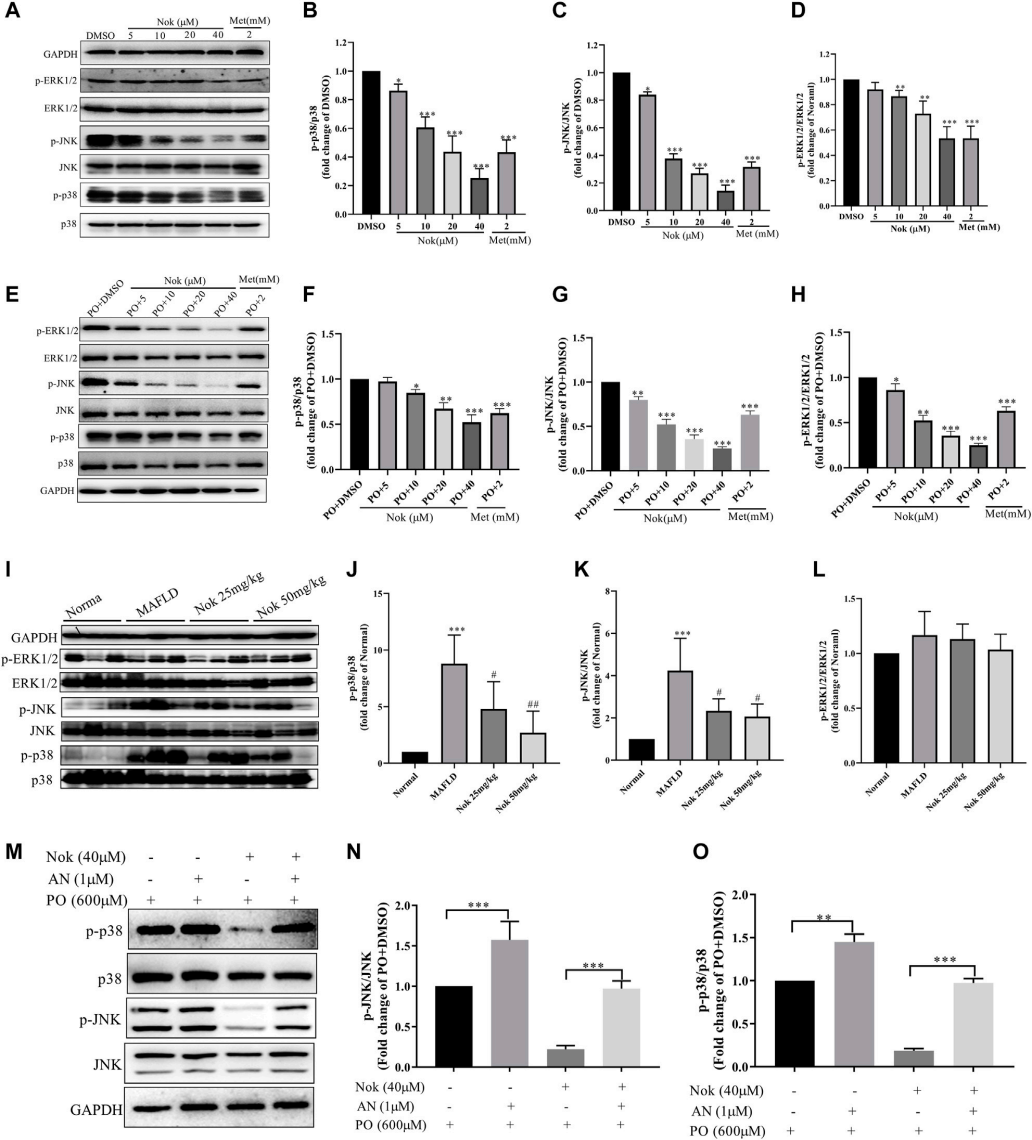
Nok inhibited MAPK signaling pathways *in vitro* and *in vivo*. Total protein was extracted from the L02 cells after Nok treatment for 24 h. The levels of p-ERK1/2, ERK1/2, p-p38, p38, p-JNK, JNK and GAPDH were determined by western blotting. Representative blots for each sample are presented in **(A)**, and fold changes in p-p38/p38 **(B)**, p-JNK/JNK **(C)** and p-ERK1/2/ERK1/2 **(D)** are shown by semiquantitative analyses. Values are the means ± SD from 3 separate experiments, *p < 0.05, **p < 0.01, ***p < 0.001 vs. DMSO. **(E)** Western blots were performed to analyze the phosphorylated and total protein levels of p38, JNK and ERK1/2 in L02 cells with Nok treatment and undergoing PO induction, and representative western blot images are shown as above. The fold changes in p-p38/p38 **(F)**, p-JNK/JNK **(G)** and p-ERK1/2/ERK1/2 **(H)** are shown by semiquantitative analyses. The values are the means ± SD from three separate experiments, *p < 0.05, **p < 0.01, ***p < 0.001 vs. PO + DMSO. After 12 weeks of Nok treatment of C57BL/6J mice with a HFD, total protein was obtained from the mouse livers. The levels of p-ERK1/2, ERK1/2, p-p38, p38, p-JNK, JNK and GAPDH were tested by western blotting. Representative blots for each group are presented in **(I)**. Fold changes in p-p38/p38**(J)**, p-JNK/JNK **(K)** and p-ERK1/2/ERK1/2 **(L)** were determined by semiquantitative analyses, and the values are expressed as the means ± SD (n = 6 per group). *p < 0.05, **p < 0.01, ***p < 0.001 vs. Normal group mice #*p* < 0.05, ##*p* < 0.01, ###*p* < 0.001 vs. MAFLD model mice. **(M)** Inhibitory effect of anisomycin (AN, a JNK agonist) on Nok inhibition of MAPK pathways, especially the JNK pathway. Representative western blot images are shown in (M), and fold changes in p-JNK/JNK **(N)** and p-p38/p38 **(O)** are shown by semiquantitative analyses, *p < 0.05, **p < 0.01, ***p < 0.001.

The MAPK signaling pathway is closely related to a variety of cellular biological processes, such as cell growth, differentiation, apoptosis, necrosis and inflammation. Excessive MAPK signaling pathway activation is commonly detected in many metabolic diseases, including MAFLD. Elevated MAPK signaling involved in the development of MAFLD and has been widely confirmed and reported. Our RNA-seq results also demonstrated that Nok might affect the MAPK signaling pathway in the liver. Our animal and cellular experiments showed that Nok inhibited MAPK pathways in hepatocytes by western blots of *in vitro* and *in vivo* samples, as shown in Figure 6. We first detected the main MAPK signaling pathway protein kinases, including ERK1/2, p-ERK1/2, p38, p-p38, JNK, and p-JNK, in L02 cells by western blotting. The results showed that Nok significantly downregulated the p-ERK1/2, p-p38, and p-JNK levels (Figure 6A) and showed no effect on the total MAPK protein kinase level, especially when the Nok concentration reached 40 μM (Figures 6A–D). Moreover, inhibition of the p38 and JNK signaling pathways was more potent and evident at only 10 μM (Figures 6A–D). The levels of p-p38 and p-JNK in the 10 μM Nok treatment group were 0.61 ± 0.040-fold and 0.37 ± 0.02-fold lower than those in the DMSO treatment group (Figures 6C,D). When the treatment concentration increased to 40 μM, the p38 and JNK signaling pathways were very significantly suppressed, and the levels decreased to 0.25 ± 0.04-fold and 0.14 ± 0.03-fold compared to DMSO (Figures 6C,D). We further detected the effects of Nok on MAPK signaling in L02 cells treated with PO and identified inhibition of MAPK signaling and downregulated levels of p-ERK1/2, p-P38 and p-JNK, as previously detected in L02 cells directly treated with Nok (Figures 6E–H). Moreover, its inhibition of MAPK signaling pathway activation was abolished by pretreatment with anisomycin, a JNK agonist (Figure 6M–O). We further detected the effects of Nok on MAPK signaling pathways *in vivo*. In NAFLD mouse liver tissues, the MAPK signaling pathways were significantly activated based on western blot analysis. The levels of p-p38 and p-JNK were potently upregulated in MAFLD model mice, but the phosphorylated levels of these primary MAPK signal pathway kinases were decreased in Nok-treated mice at doses of 25 mg kg^−1^ and 50 mg·kg-1 (Figure 6I-L).

### Anti-Inflammatory Effects of Nok

**FIGURE 7:**
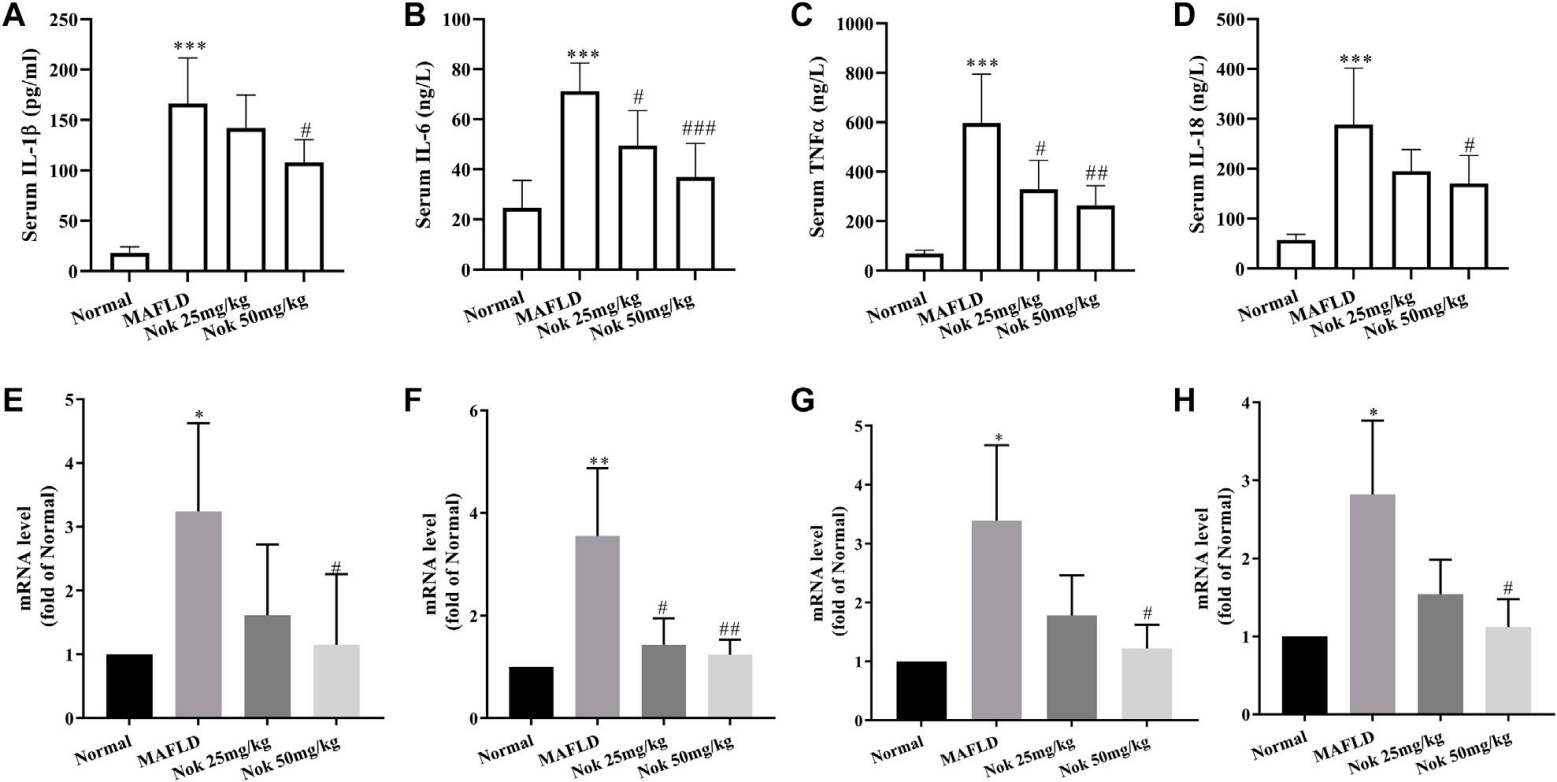
Anti-inflammatory effects of Nok. After 12 weeks of daily administration of Nok to C57BL/6J mice fed a HFD, the serum IL-1β **(A)**, IL-6 **(B)**, TNFα **(C)**, and IL-18 **(D)** levels were tested. At the same time, we detected the mRNA levels of IL-1β **(E)**, IL-6 **(F)**, TNFα **(G)**, and IL-18 **(H)** in livers. Values are the means ± SD (n = 6 per group). *p < 0.05, **p < 0.01, ***p < 0.001 vs. C57BL/6J normal mice. #*p* < 0.05, ##*p* < 0.01, ###*p* < 0.001 vs. the MAFLD model mice.

Inflammation is one of the major risk factors for the development of MAFLD. MAPK signaling pathways, especially the p38 and JNK signaling pathways, regulate the production of inflammatory cytokines. The serum levels of IL-1β, IL-18, TNFα and IL-6 are the major inflammatory cytokines that were tested in this study. The levels of all these cytokines were significantly higher in MAFLD model mice than in normal animals (Figures 7A–D), and they were significantly decreased by Nok treatment, especially at a 50 mg kg^−1^ dose (Figures 7A–D). We also detected the mRNA levels of IL-1β, IL-18, TNFα and IL-6 in the liver, and Nok decreased the transcription levels of these genes (Figures 7E–H). Therefore, Nok possessed potent anti-inflammatory capability *in vivo*, which might be associated with its inhibition of MAPK signaling.

## Discussion

Metabolic-associated fatty liver disease is a new description that differs from NAFLD, in that alcohol consumption and other liver disease factors are no longer considered (Eslam, Sanyal et al., 2020). The pathophysiological changes of MAFLD include a variety of interrelated pathological processes, such as glucose and lipid metabolism disorders, insulin resistance, lipid toxicity, inflammation, liver injury, and liver fibrosis (Lin, Huang et al., 2020; Zhong, Huang et al., 2020). MAFLD has become the most common chronic liver disease worldwide, with a prevalence of 24%, and the prevalence increases to 70–80% in obese and diabetic patients (Ciardullo and Perseghin 2021). The widespread prevalence of MAFLD has placed a huge financial burden on the healthcare system in China and around the world. With the increasing number of MAFLD patients and the lack of effective treatments, the development of effective drugs for the clinical prevention and treatment of MAFLD is greatly desired. Nok is mainly extracted from *Alpiniae Oxyphyllae* Fructus, a famous traditional Chinese medicine rich in Hainan Province. This traditional medicine has been reported to have many biological activities, including antioxidant stress, anti-inflammation, and anti-apoptosis activities (Murase, Misawa et al., 2010). In this work, we focused on the efficacy and potential mechanisms of Nok in preventing MAFLD *in vitro* and *in vivo*.

Our *in vivo* results showed that Nok significantly reversed the body weight, liver weight and liver index increases in MAFLD mice, ameliorated glucose and lipid metabolism disorders, and improved liver function and glucose tolerance damage compared to MAFLD mice. The *in vitro* results showed that Nok significantly promoted glucose consumption and decreased intracellular TG accumulation in L02 cells. We further demonstrated that this activity might contribute to its regulatory effects on the AMPK and MAPK signaling pathways both *in vitro* and *in vivo*.

AMPK is a core kinase that regulates cellular energy metabolism and balance and is also closely related to the regulation of glycolipid metabolism, which is also a target for MAFLD. AMPK activation in the liver is beneficial for glucose consumption, lipid oxidative decomposition, and body energy provision. Many AMPK agonists, such as mangiferin (Yong, Ruiqi et al., 2021), berberine (Zhu, Bian et al., 2019), and metformin (Zamani-Garmsiri, Hashemnia et al., 2021), are considered potential drugs for MAFLD treatment. The results of this study are the first to demonstrate that Nok can improve MAFLD based on activation of hepatic AMPK *in vitro* and *in vivo*.

MAPK signaling pathways participate in a multitude of processes that control and are associated with MAFLD development (Jiao, Feng et al., 2013; Lawan and Bennett 2017). The MAPK signaling pathway involves three primary pathways: ERK1/2, p38 and JNK (Lawan and Bennett 2017). The ERK1/2 signaling pathway is usually involved in the proliferation and differentiation of cells, and the p38 and JNK signaling pathways are mainly involved in the cell stress response and apoptosis (Balmanno and Cook 2009; Lawan and Bennett 2017). Many studies have reported that hepatic metabolic dysfunction might cause JNK activation and promote MAFLD development (Yan, Gao et al., 2017; Cai, Zhang et al., 2018). Moreover, the expression level of p-p38 MAPK in the MAFLD model group induced by a high-fat diet was higher than that in the normal group, and the activated p38 MAPK signaling pathway promoted the development of MAFLD by leading to insulin resistance (Zeng, Tang et al., 2014). Meanwhile, inhibiting p38 MAPK activity could ameliorate MAFLD progression in a rodent animal model (Zeng, Tang et al., 2014; Lawan and Bennett 2017; Deng, Tang et al., 2018). As extensively reported, the MAPK signaling pathway mediates the production of inflammation, oxidative stress and glycolipid metabolism disorders induced by many factors, such as hyperglycemia and hyperlipidemia, during the development of MAFLD. Many compounds, such as berberine (Li, Geng et al., 2014), bergamot polyphenols (Musolino, Gliozzi et al., 2020), baicalin (Fang, Sun et al., 2019), and isoliquiritigenin (Kim, Kim et al., 2010), can ameliorate MAFLD progression by inhibiting MAPK activity. In our study, we confirmed that Nok inhibited MAPK activity *in vivo* and *in vitro*. This is the first report that Nok showed a potential role and efficacy in treating MAFLD by inhibiting MAPK activation. Interestingly, the inhibition of p38 and JNK activity by Nok was more obvious than the inhibition of ERK1/2 signaling.

Our study also showed that Nok could improve hepatic tissue glucose tolerance and liver function in HFD-induced MAFLD mice. Impaired hepatic glucose tolerance is another cause of MAFLD development, especially in type 2 diabetic or obese patients. Improving hepatic tissue glucose tolerance can significantly ameliorate the progression of MAFLD, and it is currently a common method for the clinical treatment of MAFLD. In addition, the protective effect of Nok on liver function was also observed. Collectively, these results suggested that Nok could be beneficial for preventing and treating MAFLD.

## Conclusion

In this study, we isolated Nok from *Alpiniae Oxyphyllae* Fructus and analyzed its structure and purity. Our study is the first to show that NOK an ameliorate MAFLD *in vitro* and *in vivo*. Our data showed that Nok significantly improved the body weight and liver weight/index elevation, corrected glycolipid metabolism disorder, ameliorated glucose tolerance, decreased hepatic lipid accumulation and steatosis, and protected the liver function in HFD-induced MAFLD mice *in vivo*. Nok increased hepatocyte glucose metabolism and decreased FFA-induced intracellular TG *in vitro*. We further demonstrated that the protective mechanisms of Nok against MAFLD might be associated with AMPK signaling pathway activation and MAPK signaling pathway inhibition *in vivo* and *in vitro*. In summary, we first clarified and reported that Nok, a sesquiterpene ketone from *Alpiniae oxyphyllae* Fructus, could ameliorate MAFLD, and we provide a scientific basis for its clinical application in MAFLD treatment or prevention in the future.

## Data Availability

The datasets presented in this study can be found in online repositories. The names of the repository/repositories and accession number(s) can be found at: https://www.ncbi.nlm.nih.gov/sra/PRJNA825669.
